# On the Therapeutic Action of Arsenic

**Published:** 1846-02

**Authors:** 

**Affiliations:** Senior Physician to the Military Hospital at Versailles


"*On the Therapeutic Action of Arsenic.*—M. Boudin, senior physician to the Military Hospital at Versailles, made the following communication:—After having for a considerable period taken arsenic myself, and thus convinced myself of its safety in therapeutic doses, I, during the last five years, administered arsenic to 2947 patients of various ages, and obtained the most satisfactory results from its use, without the slightest inconvenience referable to the arsenic having occurred in any one case.

"Most of these patients were affected with marsh fevers of various types; upwards of 2000 of them had been previously treated with sulphate of quinine from one to ten times; and nearly 500 of the number had previously taken, without any benefit, more or less considerable quantities of sulphate of quinine.

"Those patients were not selected; arsenic was administered to all without distinction. Neither was the medicine given at any particular season, but I observed that it was necessary to exceed the mean dose in the summer. The medicine has further been administered in various latitudes, always on a large scale, and to patients from almost every part of the world—Senegal, Algiers, America, Syria, Italy, Corsica, the Delta of the Nile, the Rhone, &c. The result has been, that for five consecutive years, I have never had to administer sulphate of quinine in a single case.

"The duration of the treatment has been generally short; the fever has seldom resisted the first or the second dose of arsenic; relapses have been extremely rare—a circumstance which, however, I chiefly attribute to the febrifuge having been continued for eight or ten days after the cessation of the accesses.

"Comparative experiments have led me to reject all the arseniates, and employ arsenious acid exclusively. The solutions of Fowler and of Pearson take too long to prepare, and it is difficult—nay, even dangerous—to adjust their doses properly; I have therefore substituted for them a solution of one grain of arsenious acid in a pint of Water.

"It is unnecessary to add opium to this solution; the great division by the large quantity of vehicle, is the best guarantee against its irritating action. A fifth part of the above solution contains the *fifth* of a grain of arsenic, and is the medium dose. It should be administered three hours before the calculated period of the access of fever. If the antecedents of the case raise the presumption that the fever is obstinate, two other doses should be previously administered, with an interval of two hours between each. I, for a long time, gave at Marseilles, as the mean dose of arsenious acid, one or two *hundredths* of a grain of arsenious acid, and this is the dose still administered by many physicians in the south of France, and especially by some of the professors at Montpellier. I now prefer the above mentioned dose of one-fifth of a grain. It has been objected to the use of arsenic that it is a poison! Three hundred years have elapsed since Paracelsus answered —'Arsenic cures because it is a poison.' Moreover, sulphate of quinine is also a poison, but nevertheless it is one of our most valuable medicines. 'But arsenic kills in a very small dose.' Be it so; but why give doses of it which kill, when it is easy to give it in quantities that cure? Besides, other medicines, as strychnine and corrosive sublimate, &c., kill in smaller doses than arsenic does; but where is the practitioner who dreams of not prescribing them? It has been said that arsenic causes, or tends to cause, visceral enlargements, &c. This fable may be excused in persons who have no experience of its administration. It has been also objected that arsenic does not cure in every season of the year; but this is not the case unless it is administered always in one invariable dose, and above all, unless the practitioner neglects to treat decidedly, and at its commencement, those affections of the digestive organs which constitute one of the most frequent complications of ague. Finally, it is said that arsenic is useless, as quinine *always* effects a cure. It is needless to enter upon a serious refutation of this mistake. Ramazzini, Baker, and J. P. Frank, have recorded epidemics which were quite refractory to bark—for example those of 1680, 1781, and 1787. But even if bark did cure always, its high price places it beyond the reach of the poorer classes."—*Dub. Med. Press, Sept.* 24, in *New York Jour. of Med.*

